# Early atraumatic recurrent dislocation after reverse total shoulder arthroplasty despite optimal implant positioning: two case reports

**DOI:** 10.1186/s12891-025-09314-3

**Published:** 2025-12-01

**Authors:** Wen-Tung Hsieh, Yu-Cheng Lee, Chung-Hsun Chang

**Affiliations:** 1 Department of Orthopedics, National Taiwan University Hospital Hsinchu Branch, Hsinchu, Taiwan; 2 Department of Orthopedics, National Taiwan University Hospital, No.7, Chung Shan S. Rd. (Zhongshan S. Rd.), Taipei, Zhongzheng Dist. 100225 Taiwan

## Abstract

**Introduction:**

Postoperative instability following reverse total shoulder arthroplasty (RTSA) is one of the most common complications and remains a significant clinical challenge. Although implant positioning, design, and surgical technique are known to influence stability, there is no universally accepted management strategy when dislocation recurs. This study reports two rare cases of early, atraumatic recurrent dislocation after RTSA, highlighting the complexity of this complication.

**Methods and results:**

Between 2014 and 2023, 182 reverse total shoulder arthroplasties (RTSAs) were performed at our institution by a single surgeon using a consistent technique. We retrospectively identified two patients who developed recurrent dislocation. One patient underwent RTSA for an acute proximal humerus fracture, and the other for fracture malunion. Dislocation occurred at 2 and 3 weeks postoperatively, respectively, without any traumatic event. Both cases required operative reduction, as closed reduction in the emergency setting was unsuccessful. Despite upsizing to a thicker polyethylene liner, instability persisted. Ultimately, open reduction followed by immobilization in an abduction brace for six weeks was performed. During follow-up, no further dislocations occurred, and both patients retained their prosthesis with acceptable functional outcomes.

**Conclusion:**

Early recurrent dislocation can occur despite correct implant orientation and satisfactory intraoperative stability testing. In such cases, open reduction followed by prolonged immobilization may offer a viable option to avoid revision or resection arthroplasty.

## Introduction

Reverse total shoulder arthroplasty (RTSA) has gained significant popularity due to its effectiveness in treating a wide range of end-stage shoulder pathologies [1, 2]. Initially introduced by Paul Grammont in the 1980 s and approved in the United States in 2004, RTSA is now widely applied for conditions such as cuff tear arthropathy, severe glenohumeral osteoarthritis with glenoid deformity, irreparable rotator cuff tears, complex proximal humerus fractures, and posttraumatic sequelae [3, 4]. The expanding indications for RTSA have been driven by its success in pain relief and functional recovery, leading to a rapid increase in surgical procedures worldwide [5]. 

Despite these positive outcomes, RTSA is not without complications. Among postoperative issues, scapular notching is the most frequently observed radiographic finding. However, instability—although less common, reported in 1–16% of cases—remains one of the most clinically challenging complications and is a leading cause of early revision [6–9]. Instability has been linked to factors such as surgical technique, implant design, and soft tissue conditions [10–13]. Although advancements in prosthesis design and surgical technique have reduced instability rates, it continues to pose a significant concern for surgeons.

The management of RTSA instability remains controversial. While some studies report limited success with nonoperative strategies such as closed reduction, others recommend surgical interventions including thicker polyethylene inserts, glenosphere lateralization, management of glenoid bone loss, or even acromiohumeral cerclage suture augmentation [5, 14, 15]. Against this background, we describe two rare cases of early, atraumatic recurrent dislocation following RTSA. These cases are unique in that dislocation occurred despite appropriate implant positioning and satisfactory intraoperative stability testing. The purpose of this report is not to propose an optimal treatment strategy but to share clinical experience in managing a rare and challenging complication where conventional salvage options are limited.

## Methods and results

Between 2016 and 2023, we retrospectively reviewed all reverse total shoulder arthroplasties (RTSAs) performed at our institution by a single surgeon using a consistent technique. Among 182 patients, two were identified with early recurrent dislocation requiring multiple interventions.

### Case 1

A 69-year-old male (BMI 31.4) sustained a right proximal humerus fracture (Neer type III) with anterior dislocation of the humeral head following a traffic accident in December 2020. Preoperative computed tomography (CT) confirmed adequate glenoid bone stock, with a β angle of 27.3° and a reverse shoulder arthroplasty angle (RSA) angle of 35.4°. He underwent reverse shoulder arthroplasty the following day using a Zimmer Biomet prosthesis with a cemented fracture stem. A sling was applied postoperatively.

**Fig. 1:**
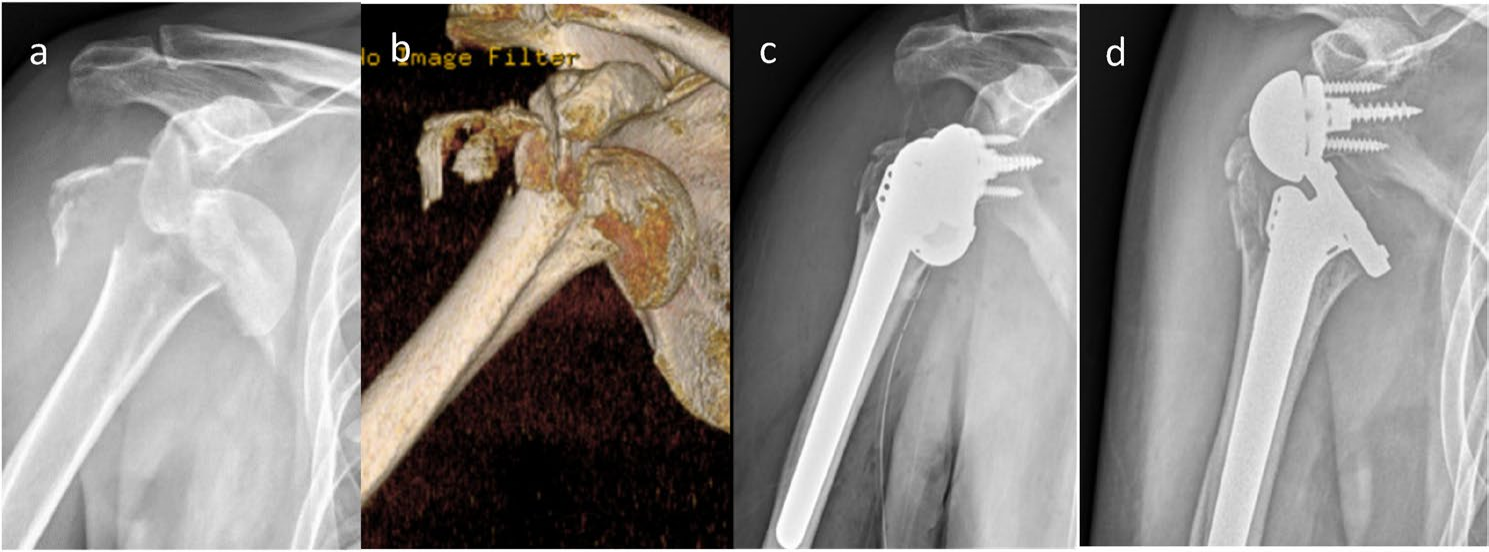
Case 1. Right proximal humerus fracture in a 69-year-old male. **a** X-ray. **b** CT scan. **c** RTSA after fracture at first time. **d** Dislocation occurring 2 weeks post-surgery

**Fig. 2:**
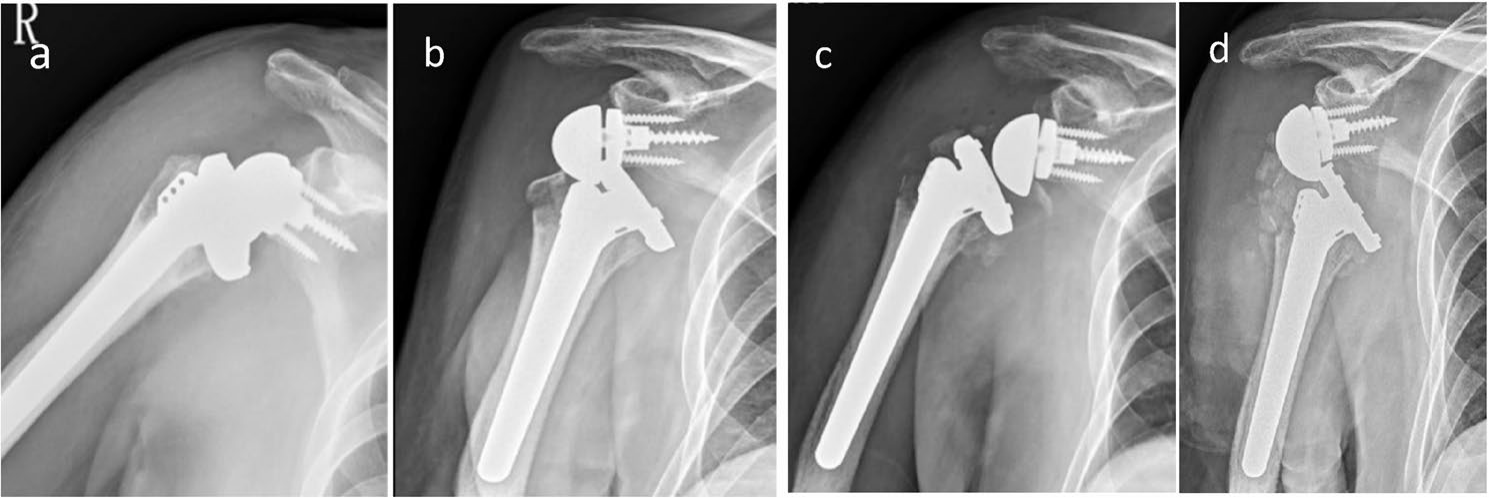
Case 1. **a** Open reduction for first time. **b** Second dislocation one month later. **c** Open reduction again and replacement of the liner with a +3mm insert. **d** Third dislocation after five days

**Fig. 3:**
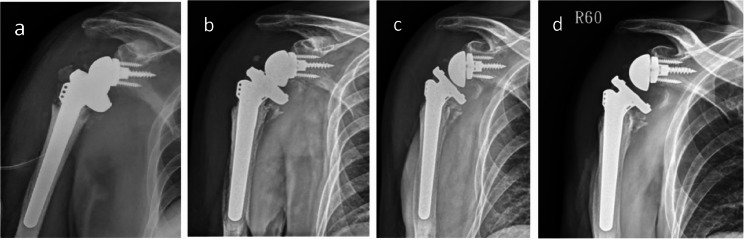
Case 1. **a** Third time open reduction. **b**, **c**, **d** X-ray after 6 months, 1 year, 1.5 years respectively

At the two-week follow-up, radiographs revealed anterior dislocation of the prosthesis without additional trauma (Fig. 1). The patient reported only mild shoulder discomfort while pulling his body using a doorknob during home exercise. Closed reduction was unsuccessful, and open reduction was performed. Despite initial stability was achieved, redislocation occurred one month later, prompting exchange to a + 3 mm polyethylene liner. A further dislocation occurred five days afterward (Fig. 2). A third open reduction was subsequently performed, followed by immobilization in neutral rotation and 30° of abduction using an abduction brace for six weeks. The brace was removed only for hygiene and supervised hand and wrist motion, followed by gradual rehabilitation (Fig. 3).

### Case 2

**Fig. 4:**
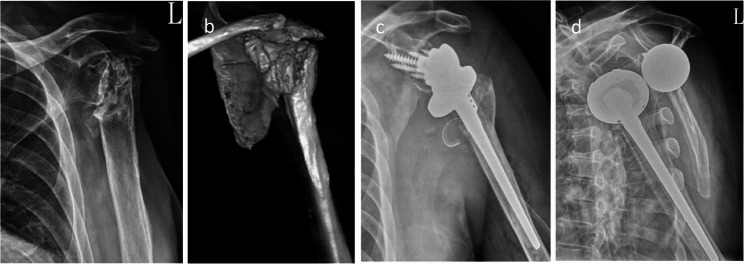
Case 2. **a**, **b** X-ray and CT scan of the proximal humerus fracture’s sequelae in a 69-year-old man. **c** RTSA at first time. **d** Dislocation after RTSA 2 weeks post-surgery

**Fig. 5:**
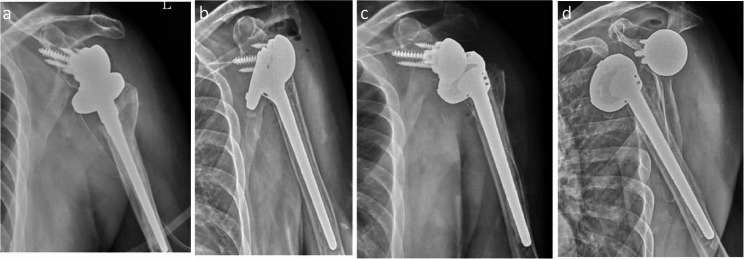
Case 2. **a** Open reduction for first time. **b** Second dislocation after two days. **c** Open reduction again and replacement of the liner with a +3mm insert. **d** Third dislocation

**Fig. 6:**
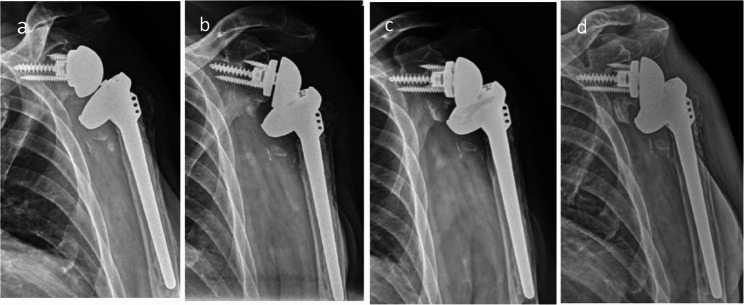
Case 2. **a**, **b**, **c**, **d** X-ray after 3 months, 6 months, 1 year, 1.5 years respectively

Another 69-year-old man (BMI 28.2) had sustained a left proximal humeral fracture in 2004, which was treated with open reduction and internal fixation, followed by implant removal eight years later. Preoperative CT showed marked glenoid deformity, precluding accurate measurement of inclination angles. In November 2021, he underwent RTSA using a Zimmer Biomet prosthesis for symptomatic chronic malunion. At the first follow-up, anterior dislocation was identified radiographically (Fig. 4). The patient denied acute pain but reported frequent use of the operated arm for stair climbing with a handrail. Open reduction was performed; however, redislocation occurred within two days. The liner was upsized to + 3 mm, yet recurrent dislocation persisted (Fig. 5). A second open reduction was followed by six weeks of immobilization in an abduction brace before progressive rehabilitation (Fig. 6).

### Implant configuration and radiographic parameters

In both cases, the humeral stem was implanted at approximately 20° of retroversion. The glenosphere version was not adjustable in our Zimmer Biomet system, which provides only a standard 36 mm concentric glenosphere with a 135° neck–shaft angle. At our institution, this is the only available configuration, and no alternative components with adjustable height or lateral offset are provided.

Postoperative radiographic analysis demonstrated an RSA angle of 4.8°, distalization shoulder angle (DSA) of 52.8°, and lateralization shoulder angle (LSA) of 85.2° in Case 1, and an RSA angle of − 1.2°, DSA of 64.2°, and LSA of 83.9° in Case 2. In Case 1, the greater tuberosity was repaired using the cow-hitch suture technique, which provided stable fixation at the time of surgery. Follow-up radiographs several months later confirmed radiographic healing of the tuberosity; however, dislocation occurred early in the postoperative period, likely unrelated to the final healing status. In Case 2, no tuberosity osteotomy was performed. The glenoid and humeral components were positioned according to standard technique, and soft-tissue tensioning was optimized within the constraints imposed by the chronic malunion.

## Outcome

In both cases, rehabilitation was initiated after six weeks of strict immobilization in an abduction brace. Although the recovery process was gradual, no further episodes of dislocation were observed during follow-up. At 18 months postoperatively, Case 1 achieved the ability to reach his forehead, with an American Shoulder and Elbow Surgeons (ASES) score of 70. Case 2, evaluated at 12 months, regained the ability to touch his nose, with an ASES score of 62. These results demonstrated satisfactory functional recovery and prosthesis retention despite the challenging course of recurrent instability (Table 1).

**Table 1:** Demographic data and clinical outcomesDemographic data and clinical outcomes

	Case 1	Case 2
Age	69	69
Gender	Male	Male
BMI	31.4	28.2
Dominant hand	Right	Right
Pathology	Fracture	Fracture sequela
Mechanism	Holding a doorknob	Pull on the stair handrail
ASES		
1Y	57	52
1.5Y	70	62
Pre-op		
RSA	35.4°	Not measurable due to malunion
Post-op		
RSA	4.8°	−1.2°
LSA	85.2°	83.9°
DSA	52.8°	64.2°

## Discussion

Although instability following reverse total shoulder arthroplasty (RTSA) is a well-recognized complication, its underlying causes and outcomes remain incompletely understood. The incidence of instability in our series was approximately 5%, which is consistent with previously reported rates ranging from 1% to 16% [8, 9]. While numerous biomechanical and clinical factors have been identified—including implant design, surgical technique, bone stock, and soft tissue balance—there is no consensus on the most effective treatment or long-term outcome for affected patients [9, 10, 16, 17]. 

Patient-specific factors play a critical role in RTSA instability. Obesity, male sex, and compromised soft tissue quality have all been associated with increased risk. Both of our patients were obese men who developed instability during routine daily activities rather than traumatic events, highlighting the potential contribution of subtle soft tissue imbalance and reduced compressive forces across the joint. Underlying pathology also influences stability: acute proximal humerus fractures and chronic fracture sequelae are known to predispose to recurrent instability. In our series, Case 1 allowed for complete subscapularis repair, whereas Case 2 had irreparable subscapularis deficiency due to long-standing malunion, underscoring the importance of soft tissue quality. The role of the subscapularis in rTSA remains debated, with studies reporting mixed results on its effectiveness in reducing instability [9, 18, 19]. Some authors have even argued against subscapularis tenotomy to lower the risk of instability and axillary nerve injury, although this may impair surgical visualization and predispose to impingement or dislocation [20]. In addition, deltoid dysfunction—often transient and neuropraxic—has also been implicated in poor outcomes, though it typically recovers over time [18]. Importantly, neither of our patients demonstrated neurological injury on nerve conduction velocity testing, suggesting that implant- and soft tissue–related factors were more likely responsible for the observed instability.

The design and orientation of the implant are also crucial for stability. Medialized designs, such as the Grammont-style prosthesis, have been associated with higher instability rates compared to lateralized designs [12]. Glenosphere size and humeral stem orientation can influence stability, with larger glenospheres and appropriate retroversion reducing dislocation risks [21]. The orientation and positioning of the glenoid component, particularly avoiding excessive medialization, are also critical for preventing scapular notching and instability. In our institution, only the Zimmer prosthesis is available, which provides a standard 135° neck-shaft angle liner and a single glenosphere size of 36 mm, thereby limiting intraoperative options. Therefore, the recurrence of dislocation in both cases suggests that liner inclination alone was unlikely the decisive factor. Additionally, using the RTSA instability flowchart proposed by Boileau, we compared the X-rays with the normal side and found no significant humeral shortening or medialization [22]. 

According to Boutsiadis et al., optimal postoperative outcomes are achieved when the DSA is 40°–65° and LSA is 75°–95° [23]. Our postoperative measurements (Case 1: DSA 52.8°, LSA 85.2°; Case 2: DSA 64.2°, LSA 83.9°) fell within or near these ranges, suggesting acceptable alignment; however, stability remained compromised. This finding reinforces that radiographic “safe zones” do not guarantee stability.

Intraoperative stability was assessed through a combination of passive range of motion, including abduction, flexion, internal and external rotation, anterior-posterior translation tests, and the tension of the conjoined tendon. The persistence of dislocation despite appropriate component sizing and careful soft tissue assessment suggests that undersizing of the implant is unlikely the primary cause.

Early dislocations are sometimes managed with closed reduction and immobilization. Success rates for this approach vary, with some studies reporting stability in up to 62% of cases managed conservatively [24, 25]. When conservative treatment fails, revision surgery is often necessary. This may involve adjusting the length of the humerus, changing the glenosphere size, or revising the implant to correct component malposition or improve soft tissue tension. In some cases, more extensive measures like the resection arthroplasty are required to achieve stability [26]. Other studies have also shown that 82% of early dislocation patients retained their RTSA. In other words, 18% of patients will undergo resection arthroplasty, resulting in poor outcomes [8]. A previous study reported that a patient who underwent conversion to hemiarthroplasty for anterior superior escape had an ASES score of only 38.8 points. Two patients who underwent resection arthroplasty had ASES scores of 41.4 and 51.6, respectively [27]. 

In our cases, both patients experienced recurrent dislocations despite upsizing the liner by 3 mm. Definitive management was achieved with open reduction and strict six-week immobilization in an abduction brace, resulting in stable shoulders and acceptable ASES scores (> 60) at follow-up. Although immobilization for six weeks may result in subsequent stiffness, this can be resolved through rehabilitation, allowing the retention of the original RTSA.

Preoperative planning and implant orientation are essential for preventing RTSA instability. CT evaluation guided implant placement. Both glenoid components were positioned with neutral to slight inferior inclination, and the humeral components were placed in approximately 20° retroversion. The neck-shaft angle (135°) and liner thickness were optimized, yet instability recurred, suggesting that patient-related factors and soft tissue imbalance contributed more significantly than implant orientation in these cases.

Several factors influence RTSA stability, including implant design, soft tissue tension, and patient characteristics. In most cases of instability, adjusting implant components or correcting soft tissue balance can restore stability. However, the two cases described here were unusual: both experienced early, atraumatic dislocations despite correct component alignment and intraoperative confirmation of stability. Collectively, these cases emphasize that early instability can occur even with apparently optimal component orientation and alignment. This highlights the unpredictable nature of this complication and the importance of strict postoperative protocols when conventional measures fail. The main limitation of this report is the very small number of cases, which precludes broad generalization of treatment strategies. Nevertheless, these cases emphasize the importance of recognizing patient- and soft tissue–related risks, and demonstrate that prolonged immobilization may be a viable option before resorting to revision or resection arthroplasty.

## Conclusion

When preoperative imaging rules out significant bony defects and intraoperative testing confirms stability, yet dislocation occurs atraumatically in the presence of normal nerve function, open reduction followed by strict six-week immobilization may help preserve the original prosthesis and avoid revision or resection arthroplasty. This report is intended to share clinical insights from our experience rather than to establish definitive treatment guidelines.

## Data Availability

This is a retrospective case report, and the data only include X-ray images and a few ASES scores. All data generated or analyzed during this study are included in this published article.
